# 2018: The translational integrator: Facilitating collaboration and bridging the “Valley of Death”

**DOI:** 10.1017/cts.2017.154

**Published:** 2018-05-10

**Authors:** Alexandra Joelle Greenberg, Nathan P. Staff, Anthony Windebank

**Affiliations:** Mayo Clinic, Rochester, NY, USA

## Abstract

OBJECTIVES/SPECIFIC AIMS: Translating conventional and regenerative medicine strategies from the research laboratory into the clinic is a complex process that can delay bringing novel therapies to the patient. Navigating the increasingly complex regulation surrounding cell-based and combination product technologies is a major challenge for the translational biomedical scientist. To this end, Mayo Clinic created a new position, the “Translational Integrator,” as part of the cGMP Biomaterials Facility in the Center for Regenerative Medicine. METHODS/STUDY POPULATION: The Translational Integrator educates investigators about FDA standards and regulatory pathways; determines where the product is on the translational spectrum; works to understand the science behind the product; determines what additional studies may be needed; supports investigators in preparing for FDA communications and submissions; and educates researchers about institutional resources and funding mechanisms needed to move their product into manufacturing and trials. A primary objective is to meet investigators at an early stage in product development to avoid conducting potentially redundant work to meet regulatory requirements. RESULTS/ANTICIPATED RESULTS: Robust training in clinical and translational research methodology enables the integrator to facilitate the collaboration necessary between investigators, clinicians, institutional resources, regulators and funders to move products towards FDA IND/IDE approval and first-in-human trials. It is an iterative process using technology/translational readiness criteria, project management and review by subject matter experts that is highly interactive and customized to each project. Current projects include topics in orthopedic surgery and ENT. In creating and refining this position, several key lessons have been learned. DISCUSSION/SIGNIFICANCE OF IMPACT: First, the Translational Integrator must undergo constant reflection and assessment of investigator needs, which requires flexibility and understanding that their role may change in the context of each product. Second, the support that the Translational Integrator provides can shift the mindset of the investigator from being averse to engaging in the translational process to eager to move their product forward. Finally, for the investigator who does not personally want to move their work into first-in-human trials, establishing connections to intellectual property generation and licensing may support movement of their findings into patients.

